# Exogenous Transforming Growth Factor-β1 and Its Helminth-Derived Mimic Attenuate the Heart's Inflammatory Response to Ischemic Injury and Reduce Mature Scar Size

**DOI:** 10.1016/j.ajpath.2023.09.014

**Published:** 2023-10-11

**Authors:** Rachael E. Redgrave, Esha Singh, Simon Tual-Chalot, Catherine Park, Darroch Hall, Karim Bennaceur, Danielle J. Smyth, Rick M. Maizels, Ioakim Spyridopoulos, Helen M. Arthur

**Affiliations:** ∗ Biosciences Institute, Faculty of Medical Sciences, Newcastle University, Newcastle, United Kingdom; † Translational Research Institute, Faculty of Medical Sciences, Newcastle University, Newcastle, United Kingdom; ‡ Wellcome Centre for Integrative Parasitology, School of Infection and Immunity, University of Glasgow, Glasgow, United Kingdom

## Abstract

Coronary reperfusion after acute ST-elevation myocardial infarction (STEMI) is standard therapy to salvage ischemic heart muscle. However, subsequent inflammatory responses within the infarct lead to further loss of viable myocardium. Transforming growth factor (TGF)-β1 is a potent anti-inflammatory cytokine released in response to tissue injury. The aim of this study was to investigate the protective effects of TGF-β1 after MI. In patients with STEMI, there was a significant correlation (*P* = 0.003) between higher circulating TGF-β1 levels at 24 hours after MI and a reduction in infarct size after 3 months, suggesting a protective role of early increase in circulating TGF-β1. A mouse model of cardiac ischemia reperfusion was used to demonstrate multiple benefits of exogenous TGF-β1 delivered in the acute phase. It led to a significantly smaller infarct size (30% reduction, *P* = 0.025), reduced inflammatory infiltrate (28% reduction, *P* = 0.015), lower intracardiac expression of inflammatory cytokines IL-1β and chemokine (C-C motif) ligand 2 (>50% reduction, *P* = 0.038 and 0.0004, respectively) at 24 hours, and reduced scar size at 4 weeks (21% reduction, *P* = 0.015) after reperfusion. Furthermore, a low-fibrogenic mimic of TGF-β1, secreted by the helminth parasite *Heligmosomoides polygyrus*, had an almost identical protective effect on injured mouse hearts. Finally, genetic studies indicated that this benefit was mediated by TGF-β signaling in the vascular endothelium.

## Graphical abstract

**Figure:**
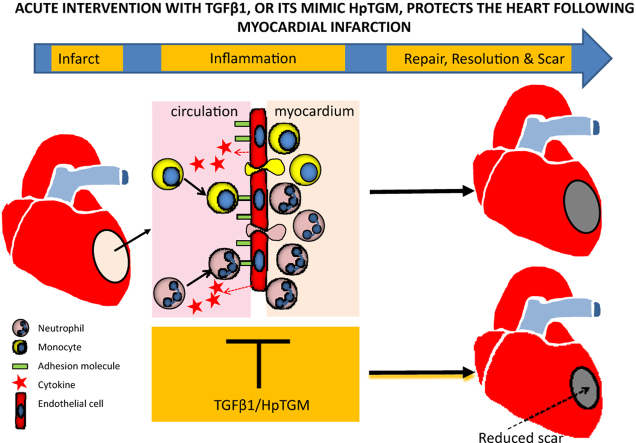

Despite major improvements using primary percutaneous intervention (PPCI) to treat patients with acute ST-elevation myocardial infarction (STEMI), progression to heart failure after infarction represents a major clinical problem.^1^^,^^2^ Despite state-of-the-art medical care, 22% of patients with STEMI treated with PPCI develop heart failure symptoms within 1 year.^3^ Detrimental progression is substantively determined by the original infarct size and time to reperfusion. An acute exuberant proinflammatory response can further enhance local cardiac injury. Over time, this can lead to adverse ventricular remodeling and gradual loss of cardiac function that can result in heart failure. For patients with STEMI, particularly those with large infarcts, additional intervention in the acute phase is needed to reduce ischemia-reperfusion injury and protect myocardial tissue, thereby reducing the risk of progression to heart failure.

The immediate effect of an acute coronary occlusion is cardiomyocyte death due to anoxia. Timely reperfusion is the most effective treatment to save ischemic myocardium. However, reperfusion itself causes release of damaging reactive oxygen species, whereas necrotic cardiomyocytes release alarmins that activate innate immune cells. Proinflammatory cytokines are up-regulated within injured myocardium, and an enhanced proinflammatory environment in the myocardium leads to further increases in immune cell infiltration associated with increased myocardial cell death.^4^^,^^5^ Therefore, dampening the immediate proinflammatory response after ischemia-reperfusion has the potential to protect the surviving myocardium from further injury.

Transforming growth factor (TGF)-β1 is an endogenous circulating and tissue-resident protein that has multiple critical roles in regulating the development and maintenance of the cardiovasculature.^6^ While it primarily circulates in a latent form, it is also stored in a latent form within tissues. It is activated by complex proteolytic mechanisms in response to tissue injury.^7^ Active TGF-β1 ligand signals via a tetrameric TGF-β receptor protein complex on the cell surface that activates SMAD2/3 transcription factors to regulate gene expression.^8^ The pleiotropic properties of TGF-β1 make it a major driver of anti-inflammatory responses.^9^, ^10^, ^11^, ^12^ In line with this property, mice lacking TGF-β1, which survive to birth, die in early postnatal life from multifocal inflammatory autoimmune disease.^13^^,^^14^ Furthermore, in a mouse model of atherosclerosis, inhibition of TGF-β signaling promotes development of atherosclerotic lesions with an increased inflammatory component.^15^ In heart tissue injured after MI, TGF-β1 is critical for promoting the transition from an early proinflammatory phase to a later proreparative phase of cardiac healing. It drives this transition via a number of mechanisms, including promoting the formation of anti-inflammatory T-regulatory cells, stimulating angiogenesis, and initiating fibrosis.^7^^,^^16^ It has additional anti-inflammatory effects on the vascular endothelium, including reducing expression of E-selectin, required for leukocyte adhesion before extravasation into the injured tissue.^17^^,^^18^ An early trigger of some of these anti-inflammatory properties is expected to be beneficial in dampening an exuberant proinflammatory response immediately after infarction. In line with this hypothesis, studies using rat and feline models of cardiac ischemia reperfusion showed that treatment with exogenous TGF-β1 in the acute phase (ie, during ischemia and before reperfusion) reduces myocardial injury within the first 24 hours of reperfusion.^19^^,^^20^ However, these studies did not investigate the more important longer-term effects on cardiac outcomes. The current study confirmed the myocardial protective role of exogenous TGF-β1 delivered in the acute phase of cardiac ischemia-reperfusion in a mouse model. Furthermore, this protection involves an early anti-inflammatory effect and leads to reduced scar size at 4 weeks after the initial injury. Because TGF-β1 also has robust profibrotic properties^21^ and therefore is unlikely to gain broad favor as a therapeutic agent, the current study investigated whether its parasite-derived mimic *Heligmosomoides polygyrus* TGM (HpTGM) has similar protective properties. HpTGM is one of a number of proteins secreted by the helminth worm *H polygyrus* to evade immune rejection from the murine gastrointestinal tract. HpTGM has no molecular homology to TGF-β1 but signals through the same TGF-β receptor complex and, importantly for this study, has greatly reduced profibrotic properties compared with TGF-β1.^22^ This work showed that a single bolus of HpTGM given at the clinically relevant time of coronary artery reperfusion has similar anti-inflammatory protective effects to TGF-β1, leading to reduced scar size.

## Materials and Methods

### Patient Study

Patients included those with STEMI (*n* = 47) from a previously published double-blind randomized controlled trial [Cyclosporin to Reduce Reperfusion Injury in Primary PCI Clinical Trial (CAPRI)]^23^ with cardiovascular magnetic resonance imaging of heart function and infarct size available at 1 week and 3 months after MI. The study complied with the principles of the Declaration of Helsinki, was approved by the local ethics committee, and obtained written informed consent from all participants (NCT02390674; *https://www.clinicaltrials.gov*). Patient details are summarized in Supplemental Table S1. Patient blood samples were taken at 15 minutes after reperfusion from the culprit coronary artery and at 24 hours after reperfusion from a peripheral vein. Serum was analyzed using a Luminex-based custom xMAP immunoassay (Bio-Rad Laboratories, Hercules, CA) with TGF-β1 detection beads.

### Mouse Surgery

All animal experiments were performed under the UK Government Animal (Scientific Procedures) Act 1986 and approved by the Newcastle University Animal Welfare and Ethical Review Body and licensed by the Home Office, UK. The study used male mice between 12 and 16 weeks of age that were of the C57BL/6 strain (purchased from Charles River Laboratories, Margate, UK). Mice with tamoxifen-induced endothelial-specific depletion of the TGF-β type II receptor [Tgfbr2fl/fl; Cdh5(BAC)-Cre-ert2] have been previously described.^24^ Tamoxifen treatment (2 mg/d) was given to adult mice (aged 10 to 12 weeks) by intraperitoneal injection for 5 consecutive days and 14 days before surgery. Surgery to induce a 60-minute transient occlusion of the left anterior descending (LAD) artery for 60 minutes was performed with the mice under isoflurane anesthesia (3% isoflurane/97% oxygen) as previously described,^25^^,^^26^ except that presurgical sedative or analgesia was provided by subcutaneous injection of midazolam (5 mg/kg) and fentanyl (0.05 mg/kg). Anethesia was confirmed at regular intervals by loss of the pedal withdrawal reflex. Sham controls had the same surgery but without coronary artery ligation. All animals entering the study were subject to the same exclusion criteria as follows: failure to recover from surgery within 2 hours, small infarct as judged by extent (<30%) of blanched left ventricular cardiac tissue at the time of ligation, no ST elevation observed on ECG during ischemic period, failure of the occluded artery to reperfuse after 60 minutes, and failed injection of drug.

### Drug Treatments

A catheter was placed in the tail vein immediately before surgery to enable time-controlled drug delivery. In the first study, recombinant murine TGF-β1 (catalog number 14-8342-82; Thermo Fisher Scientific, Hemel Hempstead, UK) was given using a dose within the range used in previous studies.^19^^,^^20^ This dose was given at 2 time points per mouse at 60 minutes apart; the first dose (33 μg/kg) was given within 1 minute of ligation of the LAD artery and the second dose (also 33 μg/kg) up to 1 minute after reperfusion of the LAD. Control mice had the same surgery but no injection. The second study used a cytokine produced by HpTGM, expressed as a recombinant protein in HEK293T cells and purified using affinity chromatography, as previously described.^22^ A single bolus of HpTGM or an equivalent volume (approximately 50 μL) of saline was given within 1 minute of reperfusion unless otherwise stated. Of note, the bioactive form of TGF-β1 is a dimer of 25 kDa, whereas HpTGM is a monomer of 49 kDa so twice the total concentration of HpTGM (132 μg/kg) was used. The surgeon (R.E.R.) was blinded to saline or HpTGM treatment.

### Area of Infarct, Density of Infiltrate, and Scar Size

Hearts were harvested at 24 hours after reperfusion, and heart cryosections (10 μm) were processed for immunofluorescent staining as previously described.^26^^,^^27^ All staining analyses were blinded to treatment group. Leukocytes were detected using rat primary antibody anti-CD45 (catalog number 103102; Biolegend, London, UK), with detection using secondary anti-rat antibodies conjugated to Alexa568 fluor. A secondary antibody-only control was used in each case to ensure immunostaining specificity. Immunostaining for leukocytes was used to characterize the density and area of immune cell infiltrate (Supplemental Figure S1).

To evaluate the extent of nonviable myocardial tissue, the heart was harvested 24 hours after reperfusion, briefly cooled, and cut into six to eight slices per heart. The rings were stained in 1% triphenyltetrazolium chloride (TTC) in 0.9% sodium chloride at 37°C for 20 minutes and fixed in 10% buffered formalin at room temperature for 1 hour. ImageJ version 1.52 (NIH, *https://imagej.nih.gov/ij*) analysis of images (taken using an MZ6 Leica microscope) of the TTC-negative (nonmetabolically active) tissue compared with the total left ventricular myocardium was used to calculate percentage of infarction. To ascertain mature scar size at 4 weeks after reperfusion, 10-μm sections of paraformaldehyde-fixed paraffin-embedded hearts were prepared using a microtome. Subsequently, every 20th section from ligature to apex through the heart (totaling a mean of 17 sections) was stained using Masson's Trichrome Stain Kit (Merck Life Science UK Ltd., Gillingham, UK) and imaged using an Aperio slide scanner. Scar size was measured using ImageJ software and presented as the percentage of total left ventricle using the method illustrated in Supplemental Figure S2.

### Intracardiac Cytokine Expression

Hearts were harvested 24 hours after reperfusion. Left ventricles were dissected, washed in phosphate-buffered saline to remove blood, harvested into RNAlater (Thermo Fisher Scientific), and frozen (−80°C) until required. Tissue was finely minced and RNA extracted using the RNeasy fibrous tissue mini kit (Qiagen, Hilden, Germany) according to the manufacturer's instructions. Random hexamers were used to prime cDNA synthesis using the Tetro cDNA synthesis kit (Merdian Bioscience, Cincinnati, OH). Cytokine expression was measured using TaqMan Universal PCR master mix and the following Taqman probes (Thermo Fisher Scientific): IL-1β: Mm00434228_m1; chemokine C-C motif ligand 2 (CCL2, also known as MCP1): Mm00441242_m1; tumor necrosis factor (TNF)-α: Mm00443258_m1 and Tgfb1 Mm01178820. Taqman probes for housekeeping genes were Mm99999915_g1 for *Gapdh* and Mm03024075_m1 for *Hprt*. Gene expression was measured in ischemia-reperfusion hearts from TGF-β1– or HpTGM-treated mice versus naive hearts using the ΔΔC_T_ comparison method.

### Cell Culture and Western Blot

Cells were derived from mice of the C57BL/6 background strain carrying the floxed *Tgfbr2* allele, tamoxifen-inducible *Rosa26-CreERT2* transgene, and the temperature-sensitive Immorto gene.^28^ Mouse coronary endothelial cells (MCECs) were isolated from heart tissue using anti-CD31–conjugated magnetic beads. MCECs were cultured in a similar way to our mouse lung endothelial cell lines^29^, ^30^, ^31^ in Promocell MV2 media with 5% serum. To deplete the *Tgfbr2* allele, cells were treated with 1 μM 4-hydroxytamoxifen for 48 hours to activate Cre-ERT2 and generate *Tgfbr2* knockout MCECs. Cells were cultured in fresh media in the absence of tamoxifen for at least an additional 48 hours before use. Western blot experiments were performed as previously described^30^ using mouse anti-pSMAD2 (diluted 1/500; catalog number 3108S; Cell Signaling Technology, Leiden, Germany) detected using anti-mouse IRDye 800CW (catalog number 925-32211; Li-Cor Biosciences, Cambridge, UK) and α-tubulin (diluted 1/2000; catalog number T6199; Merck Life Science UK Ltd., Dorset, UK) detected using anti-mouse IRDye 680RD (catalog number #925-68070; Li-Cor Biosciences). Fluorescent gel blots were imaged using an Odyssey scanner.

### Statistical Analysis

In the cohort of patients with STEMI, the primary end point was a change in infarct size at 3 months versus 1 week after PPCI. Spearman correlation analysis was used to determine the association of TGF-β1 levels at 24 hours with change in infarct size during the 3-month study period. Data were analyzed using GraphPad Prism, version 9 (GraphPad Software, San Diego, CA), and differences were considered significant at *P* < 0.05. Data were tested for normality using the Shapiro-Wilk test, where sample sizes were sufficient, and used to inform the choice of parametric or nonparametric statistical tests. Normally distributed data are presented as means ± SEM. The two-tailed *t*-test or *U*-test was used to compare two experimental groups, whereas data from multiple groups were compared using one-way analysis of variance (with Tukey post hoc corrections for multiple comparisons) or Kruskal-Wallis test (with Dunn correction).

## Results

### Circulating TGF-β1 Levels at 24 Hours After Reperfusion in Patients With STEMI Correlate with Beneficial Reduction in Infarct Size after 3 Months

**Figure 1:**
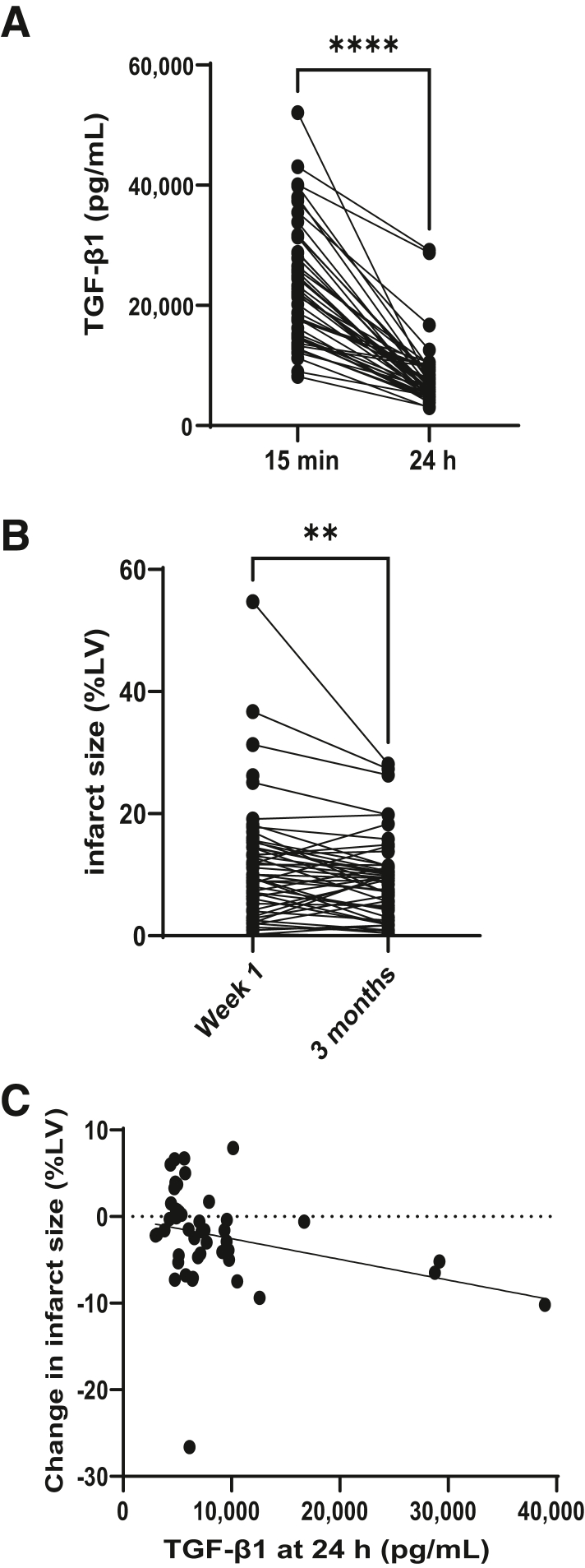
Circulating transforming growth factor (TGF)-β1 levels in patients with ST-elevation myocardial infarction (STEMI) at 24 hours after reperfusion positively correlate with a reduction in infarct size over 3 months. **A:** The median circulating level of TGF-β1 at 15 minutes and 24 hours after reperfusion in patients with STEMI. **B:** Infarct size measured by magnetic resonance between week 1 and 3 months after primary percutaneous intervention in patients with STEMI. **C:** Circulating TGF-β1 levels at 24 hours after reperfusion versus change in infarct size. Spearman's rank correlation coefficient = −0.42; *P* = 0.0034. ∗∗*P* < 0.01 (Wilcoxon two-tailed matched paired signed rank test); ∗∗∗∗*P* < 0.0001 (Wilcoxon matched paired samples test). %LV, percentage of the left ventricle.

In light of the previously reported protective role of TGF-β1 after MI in preclinical studies^19^^,^^20^ and the established role of TGF-β1 in dampening the inflammatory response,^9^, ^10^, ^11^ circulating TGF-β1 levels were examined in patients with STEMI. Data from the recent CAPRI trial, in which early blood samples and both 1-week and 3-month magnetic resonance cardiac outcome measures were available, were searched.^23^ This trial was originally designed to detect whether treatment of patients with STEMI with a single bolus of cyclosporin immediately before PPCI reduces the amount of damage to the heart compared with treatment with placebo. The study first assessed whether cyclosporin treatment affected serum TGF-β1 levels at the 2 time points tested: 15 minutes and 24 hours after reperfusion. No differences in TGF-β1 levels (Supplemental Table S2) or infarct size^23^ were observed when comparing patients with STEMI with respect to their treatment with cyclosporin or placebo. Data were therefore pooled for analysis. The median TGF-β1 level was 21,932 pg/mL at 15 minutes after reperfusion and decreased to 6248 pg/mL at 24 hours, a decrease of 3.5-fold (*P* < 0.0001) (Figure 1A). During the subsequent 3 months under standard optimal medical care, the infarct size of these patients with STEMI decreased by a mean of 2.5% (*P* < 0.01) (Figure 1B). Interestingly, circulating TGF-β1 levels at 24 hours correlated with the size of this reduction in infarct size (*P* < 0.01; Figure 1C), suggesting TGF-β1 might have beneficial effects.

### Exogenous TGF-β1 Reduces Infarct Size and Inflammatory Responses at 24 Hours After Reperfusion

**Figure 2:**
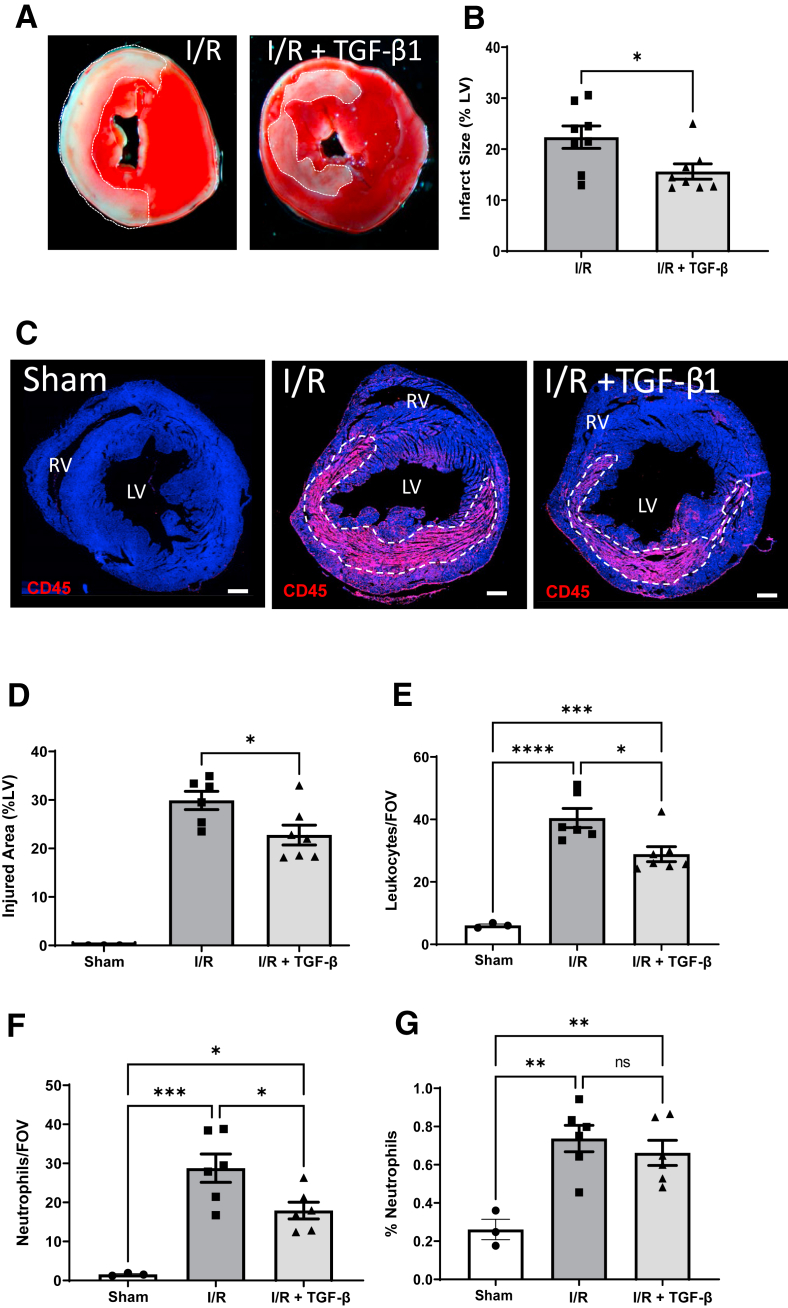
Treatment with exogenous transforming growth factor (TGF)-β1 in a mouse model of transient cardiac ischemia reduces infarct size and inflammatory infiltrate 24 hours after reperfusion. **A** and **B:** Hearts were subject to 60 minutes of infarction by transient occlusion of the left anterior descending artery followed by reperfusion with or without TGF-β1 treatment (66 μg/kg). Area of infarct as a proportion of the left ventricle (LV) was measured using viable (triphenyltetrazolium chloride) staining at 24 hours after reperfusion. Infarct area of nonviable myocardium (white) is indicated. **C:** Immunostaining of transverse heart sections with anti-CD45 (pan-leukocyte marker) at 24 hours after reperfusion was used to measure the total area of immune infiltrate (**dashed line**) expressed as percentage of the LV (%LV). This was then used as a readout of tissue injury, and this area of infiltrate is absent in sham controls. **D:** Mean values of injured area in response to TGF-β1 treatment shown for 8 images per biological replicate, taken from transverse heart sections (200 μm apart) from the intramyocardial region of the middle LV. Data analyzed by unpaired 2-tailed *t*-test (data values in shams were all zero). **E:** Anti-CD45 staining was used to measure the density of leukocyte accumulation in the injured region at 24 hours after reperfusion using the quantification methods detailed in Supplemental Figure S1. **F:** Anti-Ly6G staining was used to measure the density of myeloid cells in the injured region at 24 hours after reperfusion. **G:** The mean percentage of leukocytes that are Ly6G^+^ neutrophils in sham hearts, injured hearts, and injured hearts from TGF-β1–treated animals. Data in **D–G** were analyszd by one way analysis of variance with Tukey correction. *n* = 6 to 7 per group (**A** and **B**); *n* = 8 per group (**C**–**G**). ∗*P* < 0.05, ∗∗*P* < 0.01, ∗∗∗*P* < 0.001, ∗∗∗∗*P* < 0.0001. Scale bar = 500 μm (**C**). FOV, field of view; ns, not significant; RV, right ventricle.

**Figure 3:**
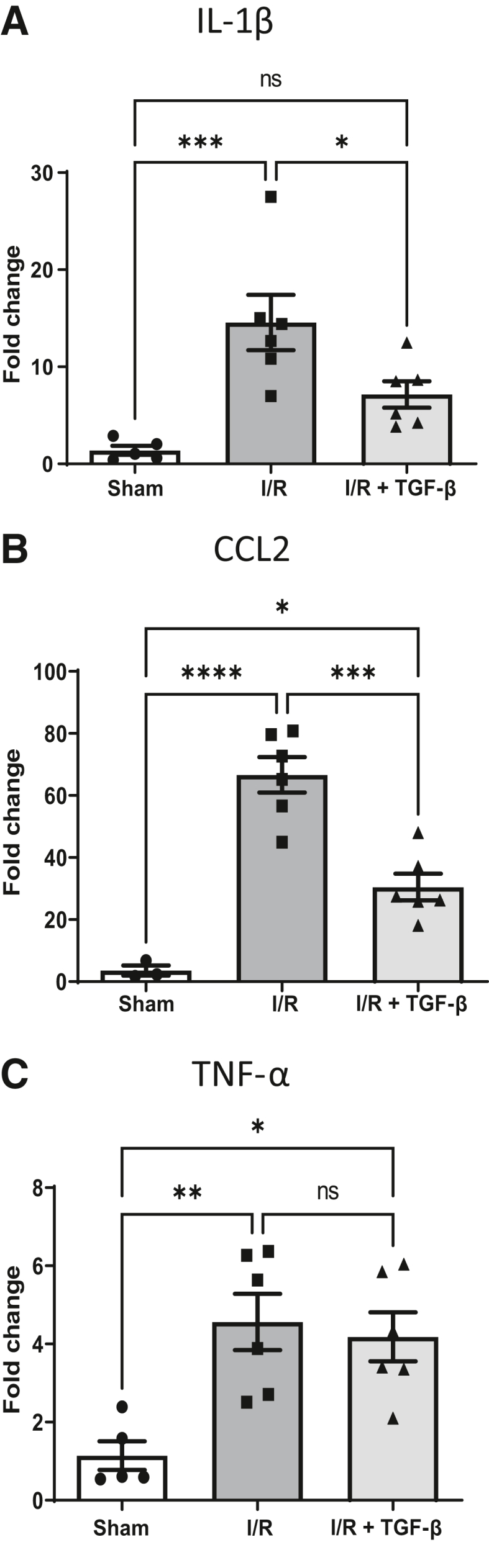
Exogenous transforming growth factor (TGF)-β1 treatment reduces myocardial-induced expression of major inflammatory cytokines. **A**–**C:** Quantitative PCR analysis of infarcted (60 minutes) left ventricular heart tissue at 24 hours after reperfusion showing cytokine IL-1β (**A**), chemokine (C-C motif) ligand 2 (CCL2) (**B**), and tumor necrosis factor (TNF)-α (**C**) compared with sham operated hearts. Gene expression data were normalized to housekeeping genes *Hprt1* and *Gapdh*, and fold change in gene expression was calculated with respect to expression in naive hearts. Data were analyzed by one-way analysis of variance with Tukey correction for multiple comparisons. *n* = 5 to 6 per group. ∗*P* < 0.05, ∗∗*P* < 0.01, ∗∗∗*P* < 0.001, and ∗∗∗∗*P* < 0.0001. ns, not significant.

The established surgical mouse model of cardiac ischemia-reperfusion was used to investigate the effect of TGF-β1 on pathologic changes in heart tissue after myocardial infarction.^25^^,^^26^ I.V. delivery of recombinant TGF-β1 at the acute phase of ischemia-reperfusion reduced infarct size at 24 hours by 30% (*P* = 0.025), based on cell viability staining with TTC (Figure 2, A and B). This TGF-β1–mediated protection corroborated results from a previous study using a cat model.^19^ Cardiac veins are the primary site of leukocyte adhesion and extravasation after reperfusion. A prior study showed that the mouse model of ischemia-reperfusion leads to significant endothelial cell leukocyte adhesion to the venous endothelium by 2 hours after reperfusion followed by a major leukocyte infiltration of the injured left ventricular tissue by 24 hours.^26^ Therefore, the effects of increased acute circulating TGF-β1 levels on the intramyocardial inflammatory infiltrate were analyzed at 24 hours after reperfusion using heart tissue immunostained for the pan-leukocyte marker CD45. TGF-β1 treatment led to a significant reduction of 23% (*P* = 0.03) in the area of leukocyte infiltrate compared with untreated ischemic hearts (Figure 2, C and D). This area of infiltrate could also be used as a readout of the injury caused by the infarct (Supplemental Figure S1), in place of the TTC assay. Importantly, the mean density of CD45^+^ leukocytes within the injured myocardium was also significantly reduced by 28% (*P* = 0.015) in TGF-β1–treated versus untreated mice (Figure 2E). Furthermore, with the use of Ly6G immunostaining to detect neutrophils, there was a significant reduction in neutrophils in the injured hearts of the TGF-β1–treated group compared with controls (Figure 2F), similar to a previous report.^19^ Moreover, the percentage of intrainfarct leukocytes that are neutrophils was not significantly different between hearts from untreated and TGF-β1–treated animals (Figure 2G). This finding suggests that the reduced infiltrate seen in the TGF-β1–treated hearts was due to a similar reduction of both neutrophils (CD45^+^Ly6G^+^; the major leukocyte cell type) and nonneutrophils (CD45^+^Ly6G^−^; the minor population). Inflammatory cytokines are known to be released rapidly within the heart after tissue injury. Intracardiac expression of three key proinflammatory mediators, TNF-α, CCL2 (also known as MCP1), and IL-1β, were all significantly up-regulated in the injured left ventricle at 24 hours after reperfusion compared with sham controls (Figure 3). In addition, the expression of CCL2 and IL-1β was reduced by >50% (*P* = 0.0004 and *P* = 0.038, respectively) in the injured left ventricle of TGF-β1–treated compared with untreated mice (Figure 3, A and B). On the other hand, induced expression of TNF-α was relatively modest and was similar in all infarcted hearts (Figure 3C).

### Exogenous TGF-β1 Treatment Reduces Mature Scar Size at 4 Weeks After Reperfusion

**Figure 4:**
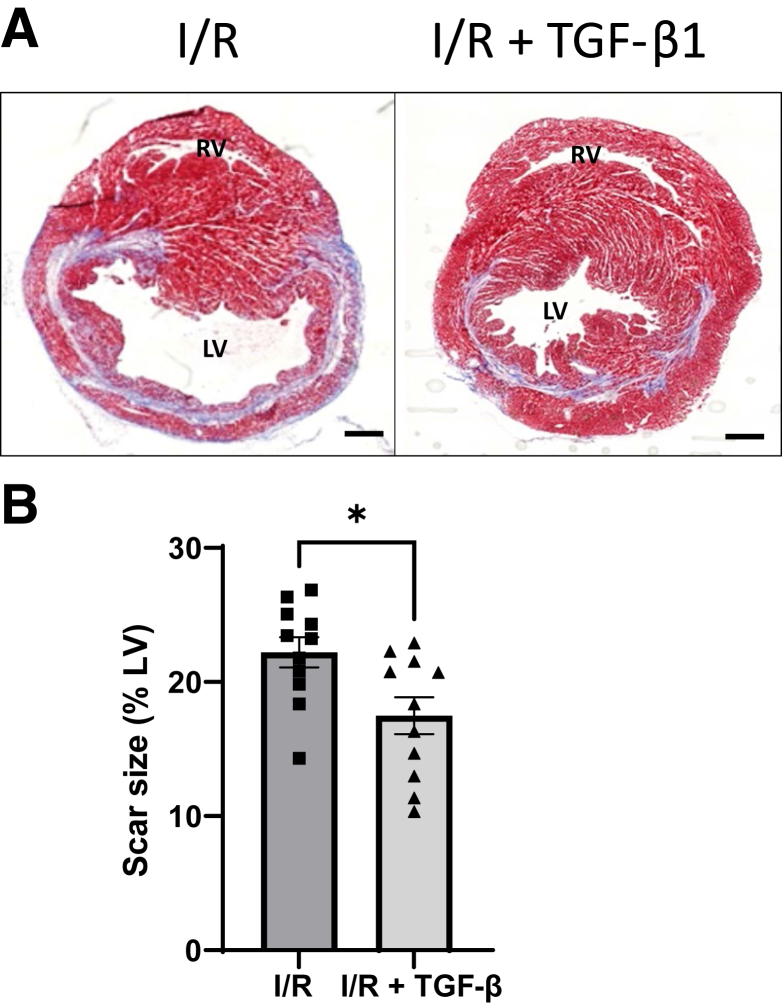
Transforming growth factor (TGF)-β1 treatment reduces scar size at 4 weeks after reperfusion. **A:** Masson's trichrome stain was used to discriminate viable muscle (pink) from collagenous scar (blue). Analysis of 17 transverse sections through each heart was used to quantify scar size as percentage of left ventricle (%LV). **B:** Scar size is significantly reduced in TGF-β1–treated mice compared with control mice subjected to the same cardiac injury of 60-minute transient ischemia. Data were analyed by unpaired *t*-test. *n* = 11 per group. ∗*P* < 0.05. Scale bars = 500 μm. LV, left ventricle; RV, right ventricle.

In this mouse model, transient cardiac ischemia due to ligation of the LAD for 60 minutes followed by reperfusion led to a mature intramural collagenous scar by 4 weeks (Figure 4A). Masson's trichrome staining discriminates viable muscle from collagenous scar and was used to measure mature scar size at 4 weeks after infarction. This intramural scar contrasts with the transmural scars that result from permanent LAD occlusion used in many other mouse studies in the literature, including our own.^32^ The mean scar size was significantly reduced by 21% in the TGF-β1–treated mice compared with untreated mice (*P* = 0.015; Figure 4B). This finding indicates a longer-term improved outcome and is consistent with the beneficial effects of short-term TGF-β1 treatment on infarct size seen at 24 hours after reperfusion. Following delivery *in vivo* exogenous TGF-β1 polypeptide has an immediate *t*_1/2_ of 11 minutes and a terminal *t*_1/2_ of 60 minutes.^33^ Therefore, most of the ligand will have cleared within 24 hours. Despite these labile properties and the observed benefit of TGF-β1 in reducing scar size, there are still likely to be concerns around the profibrotic properties of exogenously delivered bioactive TGF-β1 that might prevent its consideration as a therapeutic in patients with STEMI. Therefore the study used HpTGM, an immunomodulatory mimic of TGF-β cytokine that is produced by the helminth worm *H polygyrus*.^22^^,^^34^ HpTGM signals through the TGF-β receptor complex (Supplemental Figure S3) and mediates similar anti-inflammatory effects while lacking the robust profibrotic properties of TGF-β1.^22^

### Exogenous HpTGM Reduces Cardiac Injury at 24 Hours After Reperfusion and Decreases Mature Scar Size

**Figure 5:**
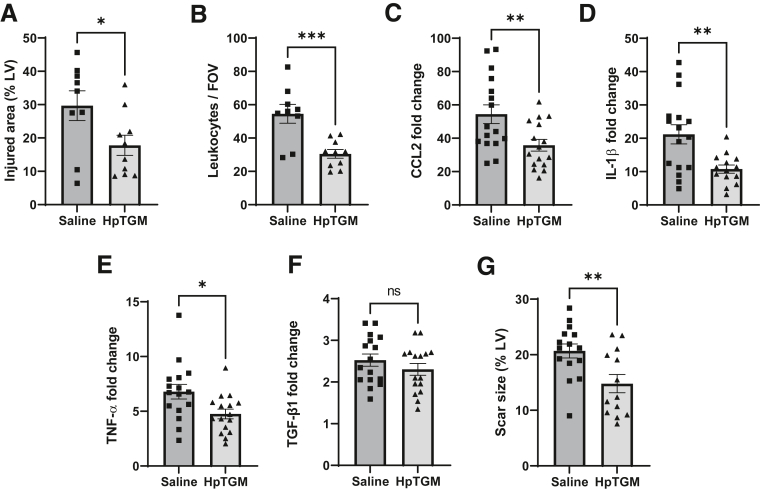
Treatment with exogenous *Heligmosomoides polygyrus* TGM (HpTGM) in a mouse model of myocardial infarction reduces infarct size, inflammatory cytokine expression, and scar size. **A:** Hearts were subject to 60-minute infarction by transient occlusion of the left anterior descending artery (LAD) followed by reperfusion with or without HpTGM treatment (132 μg/kg). Immunostaining of transverse heart sections with anti-CD45 (pan-leukocyte marker) at 24 hours after reperfusion was used to measure the total area of tissue injury. Mean values are shown for 24 images per biological replicate. Data were analyzed by unpaired two-tailed *t*-test. **B:** Anti-CD45 staining was used to measure the density of leukocyte accumulation in the injured region at 24 hours of reperfusion. Data were analyzed by unpaired two-tailed *t*-test. **C**–**F:** Quantitative PCR analysis of infarcted (60 minutes) left ventricular heart tissue at 24 hours after reperfusion shows up-regulation of cytokines chemokine (C-C motif) ligand 2 (CCL2), IL-1β, and tumor necrosis factor (TNF)-α in the left ventricle (LV) of ischemia-reperfusion (I/R) hearts from saline-treated mice compared with HpTGM-treated mice. Gene expression data were normalized to housekeeping genes *Hprt1* and *Gapdh*, and fold change in gene expression was calculated with respect to transcript levels of the target gene in naive hearts. Data were analyzed by unpaired two-tailed *t*-test. **G:** Scar size at 4 weeks after reperfusion in HpTGM-treated mice and saline-treated control mice subjected to the same cardiac injury of 60-minute transient ligation of the LAD. Data were analyzed by unpaired *t*-test. *n* = 9 to 10 per group (**A** and **B**); *n* = 14 to 16 per group (**C**–**F**); *n* = 13 to 15 per group (**G**). ∗*P* < 0.05, ∗∗*P* < 0.01, ∗∗∗*P* < 0.001. FOV field of view; %LV, percentage of left ventricle; ns, not significant; RV, right ventricle.

**Figure 6:**
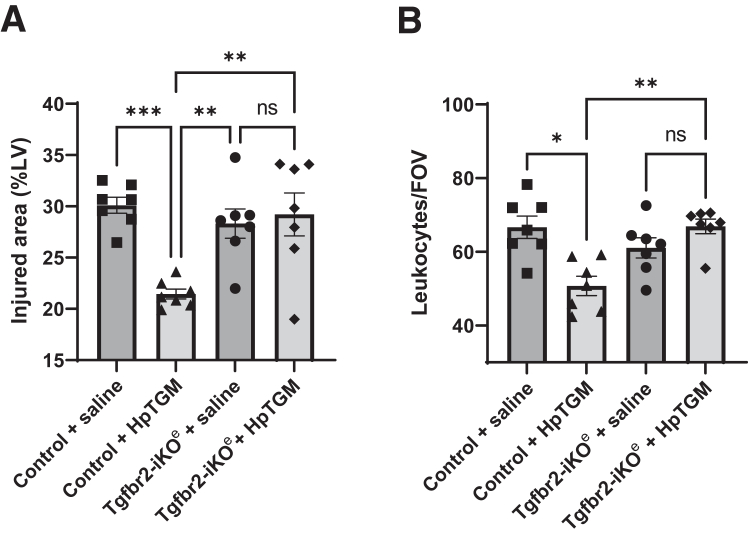
Cardiac protection of *Heligmosomoides polygyrus* TGM (HpTGM) treatment in the context of myocardial infarction is dependent on expression of transforming growth factor (TGF)-β receptor type II in endothelial cells. **A** and **B:***Tgfbr2* floxed (*Tgfbr2*^*fl/fl*^) control mice and endothelial specific *Tgfbr2* knockout (*Tgfbr2*-iKOe) mice were subject to 60-minute occlusion of the left anterior descending artery followed by reperfusion with or without HpTGM treatment (132 μg/kg). Immunostaining of transverse heart sections with anti-CD45 (pan-leukocyte marker) at 24 hours after reperfusion was used to measure the total area of heart tissue injury and the density of leukocytes per field of view (FOV). Data were analyzed by one-way analysis of variance with Tukey correction (**A**) and Kruskal-Wallis test with Dunn correction for multiple comparisons (**B**). *n* = 7 per group. ∗*P* < 0.05, ∗∗*P* < 0.01, and ∗∗∗*P* < 0.001. ns, not significant.

The treatment schedule used above was repeated in the mouse model of 60-minute transient cardiac ischemia, but with an equivalent dose of HpTGM in place of TGF-β1. The first dose was administered via the tail vein immediately before infarction and a second via the same route immediately after reperfusion. This treatment led to a very similar reduction of leukocyte infiltrate area and density in the heart at 24 hours after reperfusion (Supplemental Figure S4). This finding suggested that HpTGM had anti-inflammatory properties similar to those of TGF-β1. However, any treatment commencing at the initiation of ischemia is unlikely to be translatable to the clinic because this is before the time when most patients with STEMI reach the hospital. Therefore, the effect of a single bolus of HpTGM given intravenously (via the tail vein) immediately (<1 minute) after reperfusion was evaluated to represent a clinically relevant time point for therapeutic delivery. Control animals were subjected to the same cardiac injury and given saline, with the surgeon blinded to treatment. Single-bolus HpTGM given at the time of reperfusion reduced cardiac injury (measured using CD45 infiltrate area) by 39% (*P* = 0.038) and reduced mean leukocyte density by 44% (*P* < 0.001) compared with that in saline-treated animals (Figure 5, A and B). Similar to exogenous TGF-β1 treatment, HpTGM treatment also led to significantly reduced intracardiac expression of CCL2 (34% reduction; *P* = 0.0084) and IL-1β (49% reduction; *P* = 0.0036) compared with that in saline-treated animals (Figure 5, C and D). In addition, HpTGM appeared to be more effective than exogenous TGF-β1 in significantly reducing intracardiac expression of TNF-α (30% reduction; *P* = 0.016) (Figure 5E). Endogenous Tgfb1 transcripts were up-regulated 2.5-fold in the injured heart at 24 hours after transient ischemia, which was not altered by HpTGM treatment (Figure 5F). Importantly, the single bolus of HpTGM given at the time of reperfusion led to a significant reduction (28%; *P* = 0.0076) of mature scar size at 4 weeks after reperfusion (Figure 5G). Because the early accruement of leukocytes in the infarcted heart tissue is mediated via leukocyte extravasation through the postcapillary venules, we postulated that the TGF-β receptor in the vascular endothelium was required for the beneficial effect of HpTGM treatment. To test this, a floxed *Tgfbr2* mouse carrying a tamoxifen-activated endothelial specific Cre [*Cdh5(BAC)-Cre-ert2*] previously used in developmental studies^24^^,^^35^ was used (Figure 6). In agreement with the hypothesis, the effect of HpTGM on decreasing the size of the injured area and reducing the density of leukocyte infiltrate was completely lost after *in vivo* deletion of the TGF-β receptor *Tgfbr2,* specifically in endothelial cells. These data suggest that this early benefit of delivering i.v. HpTGM in the acute phase of MI was mediated via reduced leukocyte extravasation through the coronary vasculature.

## Discussion

Few clinical studies have examined circulating TGF-β levels in the acute STEMI setting. In stable coronary artery disease, some interesting associations have been observed between low circulating TGF-β levels and poorer outcomes.^36^ Furthermore, patients with heart failure with preserved ejection fraction have increased levels of circulating TGF-β1 compared with patients with heart failure with reduced ejection fraction.^37^ These findings indicate that increased circulating levels of TGF-β1 might be beneficial in some cardiac patient groups. In the patients with STEMI in this study, higher levels of circulating TGF-β1 at 24 hours after PPCI were significantly associated with improved scar reduction after 3 months, also suggesting TGF-β1 might provide some benefit. A likely explanation of the beneficial effects of increased circulating TGF-β1 in the acute injury phase relates to its potent anti-inflammatory properties. A mouse model of transient myocardial ischemia was used for further investigation.

Numerous preclinical studies have pointed to the benefit of an anti-inflammatory approach in reducing infarct size. For example, targeting neutrophils directly or targeting IL-1β significantly reduces infarct size.^38^, ^39^, ^40^, ^41^, ^42^ However, translating the benefits of anti-inflammatory therapy after MI to the clinic has proved challenging and led to doubts on the value of these animal studies. Nevertheless, recent clinical findings have begun to change this perception and refocus energies on understanding the benefits of anti-inflammatory therapy in patients with MI. For example, long-term anti-inflammatory therapy targeting IL1-β in patients with MI with a proinflammatory blood profile [Canakinumab Anti-Inflammatory Thrombosis Outcomes Study] reduces the risk of recurrent cardiovascular events.^43^ Furthermore, the Colchicine Cardiovascular Outcomes Trial revealed beneficial effects of early initiation of treatment after MI with the anti-inflammatory drug colchicine,^44^ which is consistent with our findings that early intervention is important to gain effective anti-inflammatory benefit. However, a simple and effective therapy at the time of PPCI to protect the remaining viable myocardium and thereby reduce the risk of progression to heart failure in patients with STEMI remains an unmet clinical need.

Dampening exuberant inflammatory responses is important also because some inflammation is beneficial and that timing of the therapeutic intervention is important. For example, early recruitment of myeloid cells to the injured heart tissue is required to remove dead cell debris. In addition, after extravasation, monocytes differentiate to macrophages that within a few days make a key transition from so-called M1 to M2 macrophage to provide key reparative proangiogenic and profibrogenic roles.^45^^,^^46^ In agreement with the anti-inflammatory protective effect of exogenous TGF-β1 in the acute phase of MI, TGF-β antagonists delivered in the acute setting amplify the inflammatory response.^47^ However, the same TGF-β antagonist treatment given later (3 to 7 days after MI) reduces adverse remodeling and myofibroblast apoptosis, consistent with a later detrimental role of TGF-β1,^47^, ^48^, ^49^, ^50^ which likely relates to its well-established role of promoting fibrosis.^51^ Thus, TGF-β1 ligand therapy after MI is beneficial only when administered within the acute phase.

Most circulating TGF-β1 is present in an inactive latent form in complex with the latent associated polypeptide and is only activated by local cellular activators, such as plasmin, thrombospondin, integrins, and proteases. In contrast, the exogenous TGF-β1 protein used in this mouse study is present in the active form and available for immediate activity. However, active TGF-β1 is very labile, with an immediate *t*_1/2_ of 11 minutes and a terminal *t*_1/2_ of 60 minutes.^33^ This indicates that, at the time of reperfusion, the majority of TGF-β1 has already been cleared. This also has implications for the profibrotic intracardiac effects that normally initiate approximately 3 days after reperfusion in rodents (and slightly later in human). Furthermore, the absence of long-term profibrotic effects in our study is consistent with the significantly reduced scar size observed after exogenous TGF-β1 treatment in the acute phase of MI. Nevertheless, to further reduce the risk of profibrotic adverse effects, the TGF-β1 mimic HpTGM, which has reduced profibrotic properties,^22^ was assessed for efficacy. Indeed, when delivered at the time of reperfusion, the anti-inflammatory and myocardial salvage effects of HpTGM are strikingly similar to those of TGF-β1. Furthermore, although HpTGM does not share molecular homology with TGF-β1, it signals through the same TGF-β receptor complex. Interestingly, the early anti-inflammatory effects of HpTGM treatment appear to be mediated by the vascular endothelium because mice without endothelial TGF-β type II receptor showed no detectable protective response to HpTGM compared with controls (Figure 6).

The importance of the vascular endothelium in myocardial ischemic injury over and above the culprit occluded vessel(s) is widely recognized.^52^ TGF-β signaling in the vascular endothelium is required for mediating the early anti-inflammatory response to exogenous HpTGM treatment. This finding is consistent with the ability of TGF-β1 to reduce vascular endothelial expression of E-selectin, a protein that mediates leukocyte adhesion to the endothelium before extravasation.^17^^,^^18^ At 24 hours after reperfusion, neutrophils predominate the inflammatory infiltrate, and TGF-β1 inhibits transmigration of neutrophils through activated vascular endothelial cells.^53^ Furthermore, intracardiac CCL2 plays a critical role in recruiting CCR2-expressing monocytes from the circulation, and SMAD3 (a key transcription factor activated by the TGF-β receptor complex) mediates inhibition of CCL2 expression.^10^ The current study was unable to discriminate the cell-specific source of the cytokines that are reduced after TGF-β1 or HpTGM treatment. However, their observed effects in reducing the injured area and the numbers of extravasated leukocytes in the myocardium by 24 hours after injury would inevitably reduce the levels of leukocyte-expressed cytokines within the infarcted heart tissue. Although the current study is limited by the lack of longitudinal cardiac function data, this early anti-inflammatory effect of short-term TGF-β1 or HpTGM treatment is substantial and clearly results in a reduced scar size at 4 weeks, consistent with a longer-term benefit of treatment.

Infarct size has a major impact on adverse cardiac remodeling, with larger infarcts leading over time to left ventricular dilatation, reduced end systolic volume, and increased risk of heart failure. Thus, significant reductions in infarct size, such as reported here, can reduce the risk of progression to heart failure. The significant levels of cardiac protection by TGF-β1 and its mimic seen here are in stark contrast to our previous efforts to save infarcted myocardium by promoting angiogenesis. Although substantial proangiogenic outcomes in the infarcted mouse heart were generated using cardiosphere-derived cell therapy, these cells failed to deliver long-term benefit in heart function.^27^ On the other hand, it is important to consider the preclinical model being used.^54^ Models of permanent myocardial infarction, in which the ischamic myocardium is almost entirely replaced by scar tissue, leave little opportunity to rescue viable tissue. In contrast, our current model of ischemia reperfusion (as used in this study) represents a better preclinical model to evaluate therapies for patients with STEMI undergoing PPCI. However, a notable limitation of this study is the use of healthy young mice. To move closer toward the multicomorbidities found in patients with MI, the model needs to be further improved by using older mice with atherosclerotic disease, for example, *ApoE* mice aged >12 months. Furthermore, antiplatelet therapy with aspirin and a P2Y12 inhibitor, often in combination with a glycoprotein IIb/IIIa inhibitor, forms part of standard patient care after PPCI. This care reduces the risk of future thrombotic events, but these drugs also have anti-inflammatory properties. Therefore, inclusion of antiplatelet drugs should be considered for inclusion in future studies to better align the rodent models with the clinical situation.

TGF-β1 has well-established anti-inflammatory properties,^9^, ^10^, ^11^ whereas HpTGM is a parasitomimetic with great clinical potential. For example, recent work shows that delivery of HpTGM has a major anti-inflammatory effect in mouse models of colitis or airway inflammation.^55^, ^56^, ^57^ The current study shows that exogenous delivery of HpTGM at the time of coronary artery reperfusion dampens the proinflammatory response of coronary endothelial cells and reduces cardiac injury, leading to increased myocardial salvage and reduced scar size with the corollary of improved prospects for long-term cardiac function. These findings strongly support future work to further investigate the protective potential of HpTGM after cardiac infarction.

## Disclosure Statement

None declared.

## Supplemental Data

Supplemental Table S2

Supplemental Figure S1 Methods used to measure area and density of leukocyte infiltrate at 24 hours after reperfusion. **A:** Transverse section of infarcted mouse heart tissue immunostained for the pan-leukocyte marker CD45 (red). Area of infiltrate (**blue line**) and loss of myocyte autofluorescence (green) were used to determine the injured area as a percentage of left ventricular wall. Intrainfarct images were taken from 8 random fields of view per section (represented in this image as **white squares**). **B** and **C:** Mean density of CD45^+^ leukocytes (**B**) and Ly6G^+^ neutrophils (**C**) per field of view within the injured myocardium of the heart was determined using eight random fields of view per section and from three sections per heart from the midinfarct region, giving a total of 24 counts per heart. Nucleated (DAPI positive) CD45^+^ or Ly6G^+^ cells were counted for each image by investigator blinded to treatment, and counts were verified by an independent observer (R.E.R. and E.S.). Scale bars: 500 μM (**A**); 20 μM (**B** and **C**). Original magnification, ×40 (**A**). LV, left ventricle; RV, right ventricle.

Supplemental Figure S2 Method to calculate scar size at 4 weeks after injury. Masson's trichrome stained transverse heart section showing viable myocardial tissue in red and collagen in blue. The area of the left ventricular wall in each section was calculated by subtracting the left ventricular lumen area lined by the endocardium (**inner black dashed line**) from the total left ventricular area (**outer black dashed line**). The scar area (**yellow line**) was expressed as a proportion of the total left ventricular wall area. A mean of 17 equidistant sections spanning from the site of the ligature to the apex were used to calculate a mean scar size for each heart. Scale bar = 200 μm. LV, left ventricle; RV, right ventricle.

Supplemental Figure S3 *Heligmosomoides polygyrus* TGM (HpTGM) signals through the transforming growth factor (TGF) receptor complex. Mouse coronary endothelial cells (MCECs) were cultured from the *Tgfbr2fl/fl;Rosa-Cre-ERT2* mouse line. Transient addition of 4-OH hydroxytamoxifen *in vitro* for 72 hours was used to activate Cre-ERT2 to generate *Tgfbr2* knockout (KO) MCECs. Non–tamoxifen-treated MCECs were used as controls. **A:** Western blot analysis shows that HpTGM (10 ng/mL) and TGF-β1 (5 ng/mL) treatments for 30 minutes leads to similar phospho-SMAD2 responses. α-Tubulin was used as a gel-loading control protein. **B:** Loss of the TGFBR2 protein in *Tgfbr2* KO MCECs leads to loss of SMAD2 phosphorylation in response to either TGF-β1 or HpTGM treatments. **C:** Tgfbr2 expression analyzed using Taqman quantitative PCR shows efficient loss of Tgfbr2 expression in *Tgfbr2* KO MCECs compared with control cells. *Tgfbr2* expression was measured using Taqman probe Mm03024091-m1, and data were normalized using housekeeping genes *Gapdh* (Mm99999915_g1) and *Hprt* (Mm03024075_m1). Gene expression in *Tgfbr2* KO cells was compared with control cells using the ΔΔC_T_ comparison method. *n* = 3 (**A** and **B**); *n* = 4 (**C**).

Supplemental Figure S4 Two bolus treatment with *Heligmosomoides polygyrus* TGM (HpTGM) leads to reduced heart injury after ischemia reperfusion Anti-CD45 staining was used to measure the density and area of leukocyte accumulation in the injured region at 24 hours reperfusion after 60 minutes of ischemia as described in Supplemental Figure S1. **A** and **B:** Two bolus treatment with TGM leads to reduced tissue injury (**A**) and reduced leucocyte density (**B**), similar to the effect of a two-bolus transforming growth factor (TGF)-β1 treatment. Data analyzed by one-way analysis of variance and include data from Figure 1, D and E to enable comparison of treatment effects. ∗*P* < 0.05, ∗∗*P* < 0.01, ∗∗∗*P* < 0.001, and ∗∗∗∗*P* < 0.0001. ns, not significant.
