# Supplementary material for: Exogenous Transforming Growth Factor-β1 and Its Helminth-Derived Mimic Attenuate the Heart's Inflammatory Response to Ischemic Injury and Reduce Mature Scar Size
Source: Am J Pathol. 2023 Oct 11;194(4):562–73. doi: 10.1016/j.ajpath.2023.09.014 (PMC12178337; doi:10.1016/j.ajpath.2023.09.014)
Supplement: Supplemental Table S1 [file mmc1.docx]

| **Supplemental Table S1. Patient characteristics (n=47)** | | |
| --- | --- | --- |
| **Demographics** |  |  |
| Male sex | 39 (83.0) |  |
| Age (years) | 65.5±10.8 |  |
| BMI, kg/m^2^ | 27.7±6.1 |  |
| **Comorbidities** |  |  |
| Diabetes mellitus | 4 (8.5) |  |
| Hypertension | 8 (17.0) |  |
| Hyperlipidemia | 4 (8.5) |  |
| eGFR | 81 (23) |  |
| **MI characteristics** |  |  |
| Onset-to-reperfusion (minutes) | 179 (147) |  |
| admission hs-cTnT | 49 (63) |  |
| 12h. hs-cTnT | 3415 (4596) |  |
| Continuous variables are presented as mean±SD or median (IQR) and categorical variables as absolute count (percentage).  Abbreviations: *BMI*, body mass index; *eGFR*, estimated glomerular filtration rate; *hs-cTnT*, high-sensitivity cardiac troponin T. | | |
