# Supplementary material for: Exogenous Transforming Growth Factor-β1 and Its Helminth-Derived Mimic Attenuate the Heart's Inflammatory Response to Ischemic Injury and Reduce Mature Scar Size
Source: Am J Pathol. 2023 Oct 11;194(4):562–73. doi: 10.1016/j.ajpath.2023.09.014 (PMC12178337; doi:10.1016/j.ajpath.2023.09.014)
Supplement: Supplemental Table S2 [file mmc2.docx]

**Supplemental Table S2**

Comparison of circulating TGFbeta1 concentrations between cyclosporin-treated and placebo control groups using independent samples Mann Whitney test

| Readout | Treatment Group | N | Median value (pg/ml) | Difference between medians | P value (two tailed unpaired Mann Whitney test) |
| --- | --- | --- | --- | --- | --- |
| TGFb1-15min | Control | 25 | 23,875 | 5,111 | 0.3 |
|  | Cyclosporine | 22 | 18,764 |  |  |
| TGFb1  24h | Control | 25 | 6,423 | 663 | 0.66 |
|  | Cyclosporine | 22 | 5,760 |  |  |
