# Supplementary figures and images for: Exogenous Transforming Growth Factor-β1 and Its Helminth-Derived Mimic Attenuate the Heart's Inflammatory Response to Ischemic Injury and Reduce Mature Scar Size
Source: Am J Pathol. 2023 Oct 11;194(4):562–73. doi: 10.1016/j.ajpath.2023.09.014 (PMC12178337; doi:10.1016/j.ajpath.2023.09.014)

Supplemental Figure S1

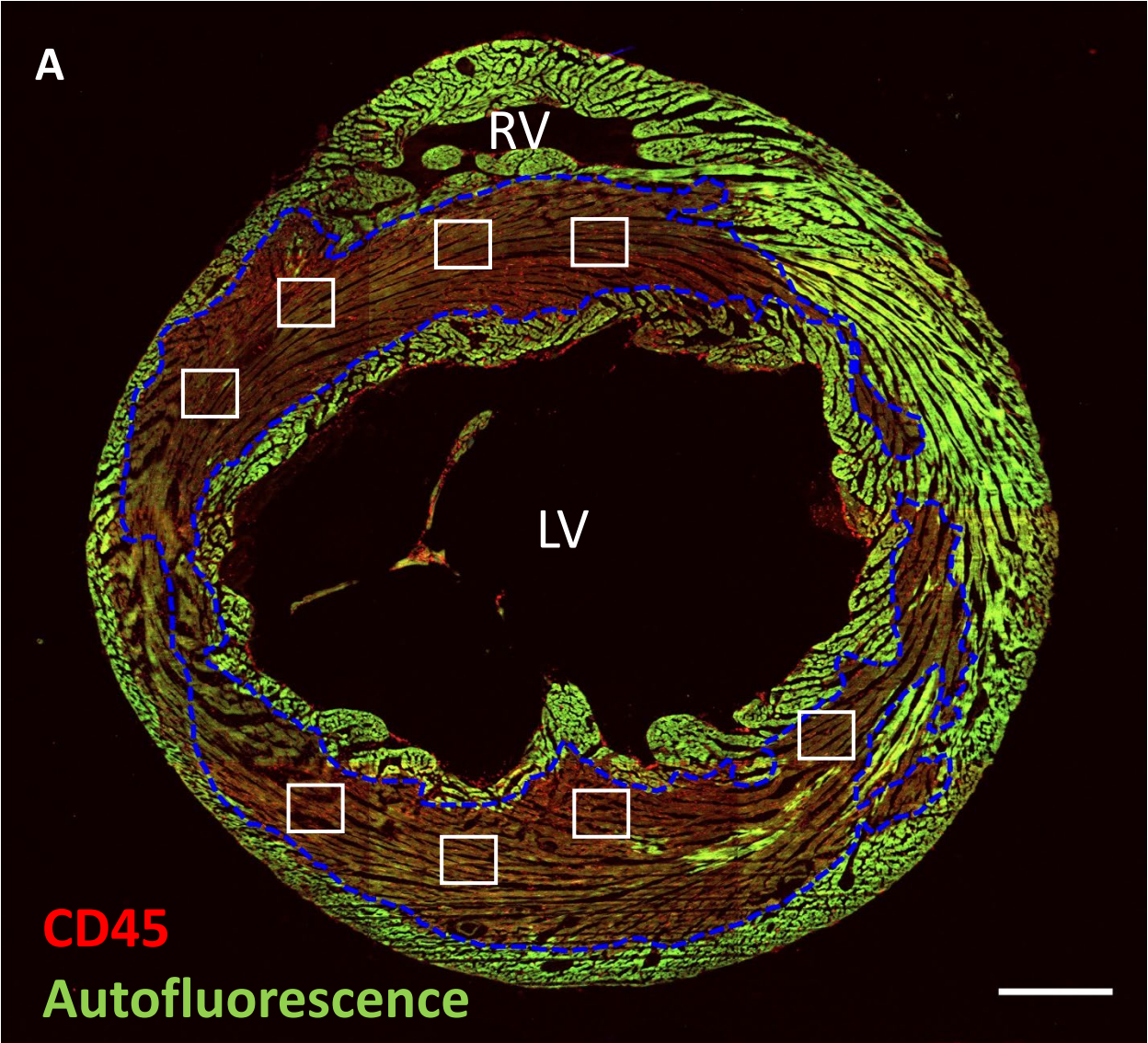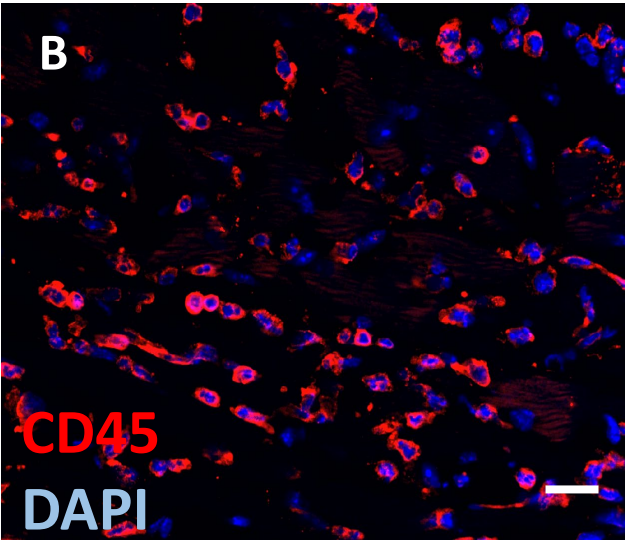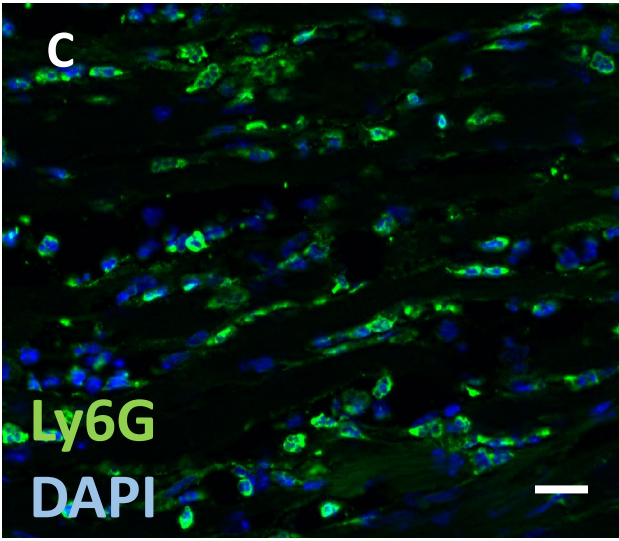

Supplement: Supplemental Figure S1 — Methods used to measure area and density of leukocyte infiltrate at 24 hours after reperfusion. A: Transverse section of infarcted mouse heart tissue immunostained for the pan-leukocyte marker CD45 (red). Area of infiltrate (blue line) and loss of myocyte autofluorescence (green) were used to determine the injured area as a percentage of left ventricular wall. Intrainfarct images were taken from 8 random fields of view per section (represented in this image as white squares). B and C: Mean density of CD45+ leukocytes (B) and Ly6G+ neutrophils (C) per field of view within the injured myocardium of the heart was determined using eight random fields of view per section and from three sections per heart from the midinfarct region, giving a total of 24 counts per heart. Nucleated (DAPI positive) CD45+ or Ly6G+ cells were counted for each image by investigator blinded to treatment, and counts were verified by an independent observer (R.E.R. and E.S.). Scale bars: 500 μM (A); 20 μM (B and C). Original magnification, ×40 (A). LV, left ventricle; RV, right ventricle. [file mmc3.pdf]

Supplemental Figure S2

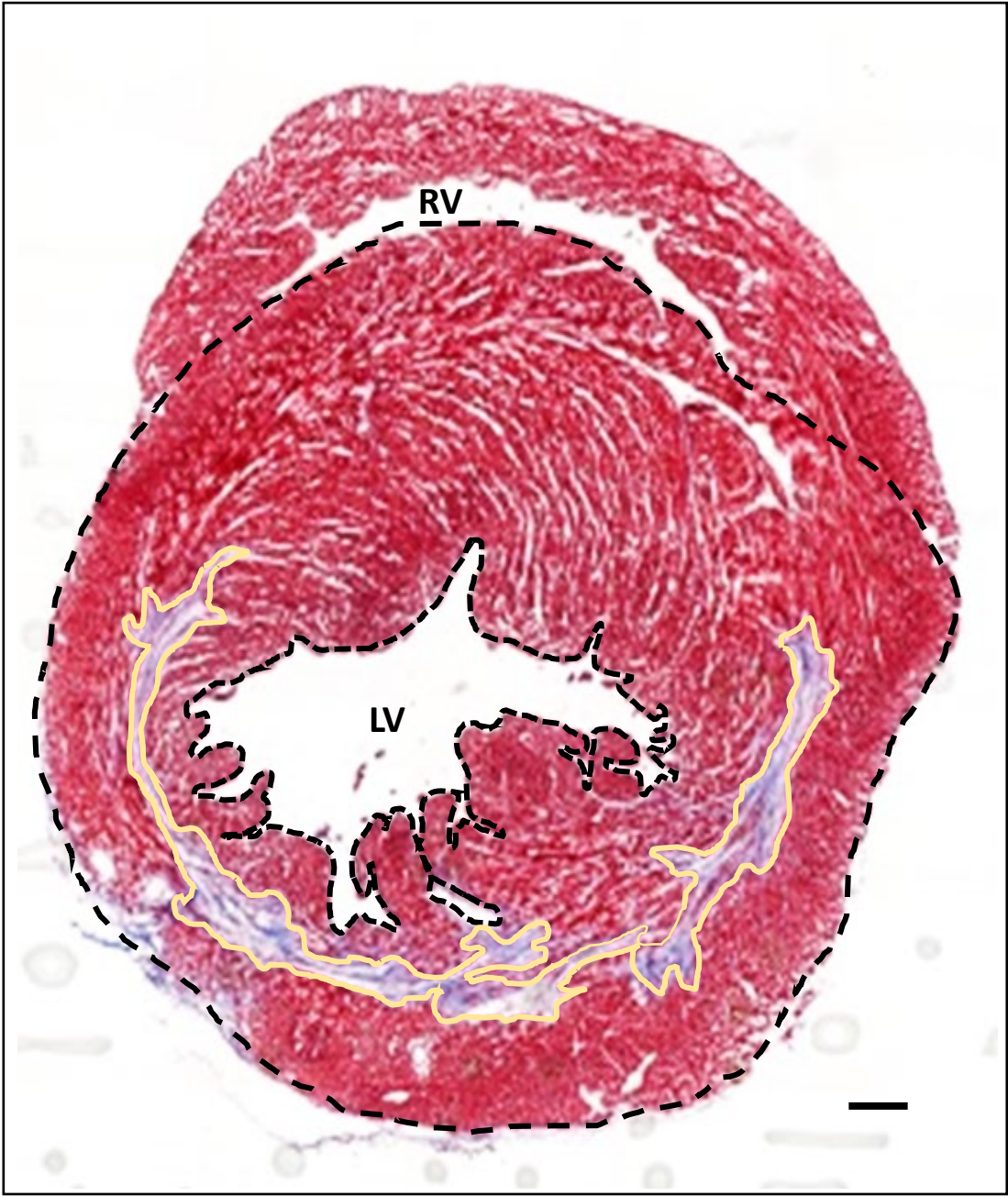

Supplement: Supplemental Figure S2 — Method to calculate scar size at 4 weeks after injury. Masson's trichrome stained transverse heart section showing viable myocardial tissue in red and collagen in blue. The area of the left ventricular wall in each section was calculated by subtracting the left ventricular lumen area lined by the endocardium (inner black dashed line) from the total left ventricular area (outer black dashed line). The scar area (yellow line) was expressed as a proportion of the total left ventricular wall area. A mean of 17 equidistant sections spanning from the site of the ligature to the apex were used to calculate a mean scar size for each heart. Scale bar = 200 μm. LV, left ventricle; RV, right ventricle. [file mmc4.pdf]

Supplemental Figure S3

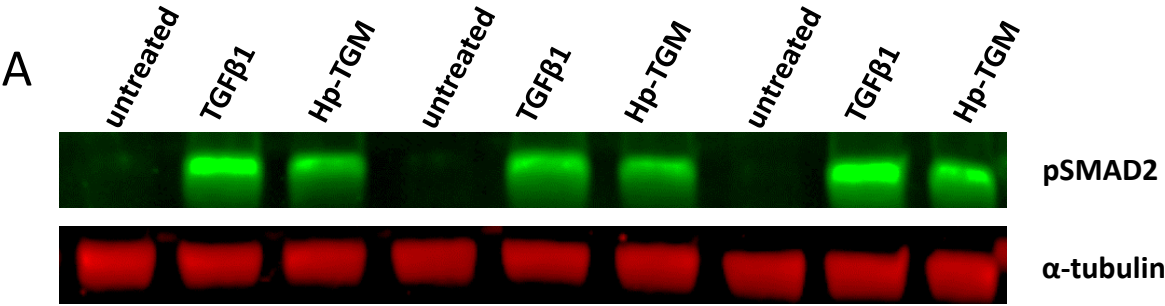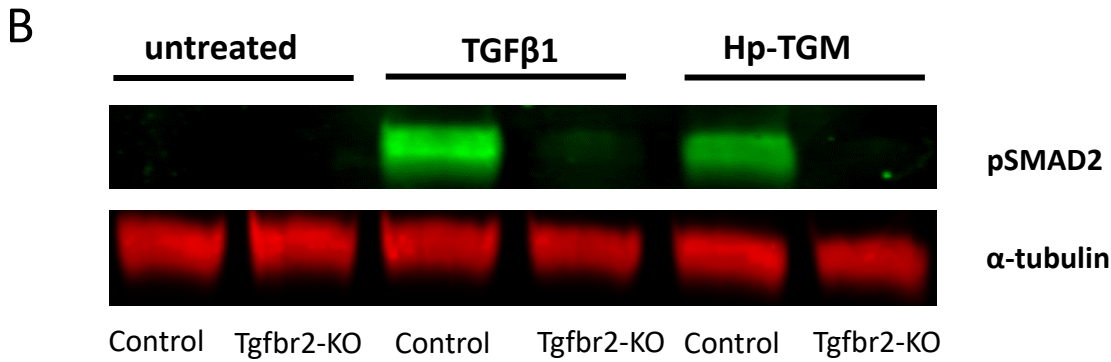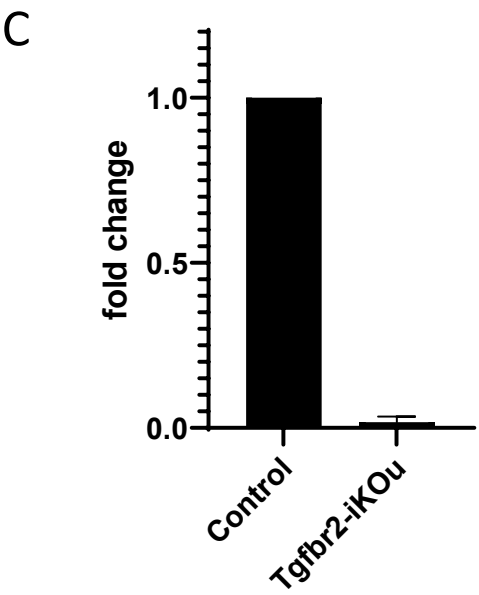

Supplement: Supplemental Figure S3 — Heligmosomoides polygyrus TGM (HpTGM) signals through the transforming growth factor (TGF) receptor complex. Mouse coronary endothelial cells (MCECs) were cultured from the Tgfbr2fl/fl;Rosa-Cre-ERT2 mouse line. Transient addition of 4-OH hydroxytamoxifen in vitro for 72 hours was used to activate Cre-ERT2 to generate Tgfbr2 knockout (KO) MCECs. Non–tamoxifen-treated MCECs were used as controls. A: Western blot analysis shows that HpTGM (10 ng/mL) and TGF-β1 (5 ng/mL) treatments for 30 minutes leads to similar phospho-SMAD2 responses. α-Tubulin was used as a gel-loading control protein. B: Loss of the TGFBR2 protein in Tgfbr2 KO MCECs leads to loss of SMAD2 phosphorylation in response to either TGF-β1 or HpTGM treatments. C: Tgfbr2 expression analyzed using Taqman quantitative PCR shows efficient loss of Tgfbr2 expression in Tgfbr2 KO MCECs compared with control cells. Tgfbr2 expression was measured using Taqman probe Mm03024091-m1, and data were normalized using housekeeping genes Gapdh (Mm99999915_g1) and Hprt (Mm03024075_m1). Gene expression in Tgfbr2 KO cells was compared with control cells using the ΔΔCT comparison method. n = 3 (A and B); n = 4 (C). [file mmc5.pdf]

Supplemental Figure S4

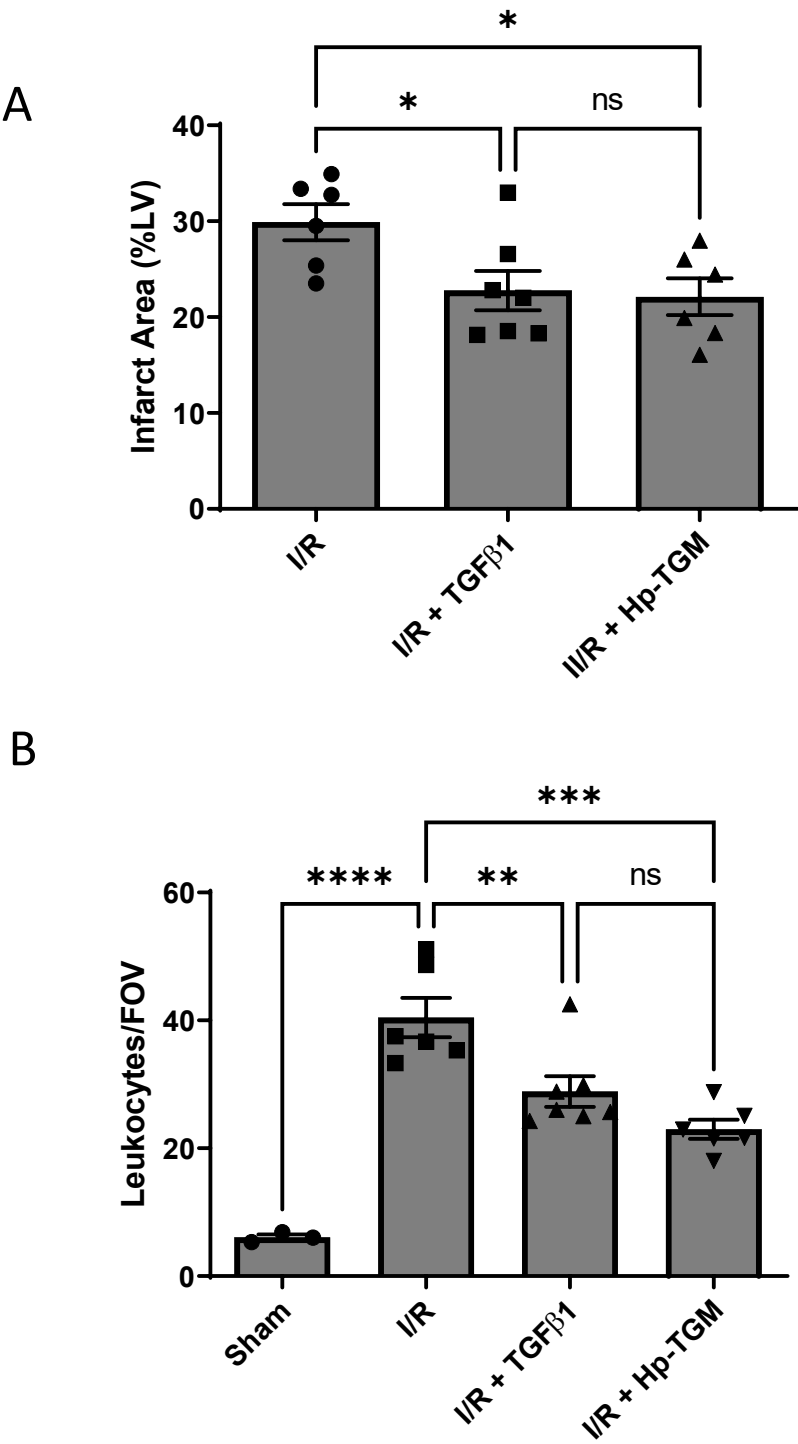

Supplement: Supplemental Figure S4 — Two bolus treatment with Heligmosomoides polygyrus TGM (HpTGM) leads to reduced heart injury after ischemia reperfusion Anti-CD45 staining was used to measure the density and area of leukocyte accumulation in the injured region at 24 hours reperfusion after 60 minutes of ischemia as described in Supplemental Figure S1. A and B: Two bolus treatment with TGM leads to reduced tissue injury (A) and reduced leucocyte density (B), similar to the effect of a two-bolus transforming growth factor (TGF)-β1 treatment. Data analyzed by one-way analysis of variance and include data from Figure 1, D and E to enable comparison of treatment effects. ∗P < 0.05, ∗∗P < 0.01, ∗∗∗P < 0.001, and ∗∗∗∗P < 0.0001. ns, not significant. [file mmc6.pdf]
